# Chemotherapy Response Score and Survival Outcomes After Neoadjuvant Chemotherapy in High-Grade Serous Tubo-Ovarian Carcinoma: A Multicenter Retrospective Study

**DOI:** 10.3390/medicina62091688

**Published:** 2026-09-03

**Authors:** Bugra Oztosun, Zehra Sucuoglu Isleyen, Zeynep Alaca Topcu, Atakan Topcu, Engin Erdemoglu, Melih Simsek, Mahmut Gumus

**Affiliations:** 1 Department of Medical Oncology, Mersin City Training and Research Hospital, Mersin 33240, Turkey; 2 Department of Medical Oncology, School of Medicine, Bezmialem Vakıf University, Istanbul 34093, Turkey; 3 Department of Medical Oncology, Istanbul Medeniyet University, Prof. Dr. Suleyman Yalcin City Hospital, Istanbul 34736, Turkey; 4 Department of Medical Oncology, School of Medicine, Istanbul Medipol University, Istanbul 34718, Turkey; 5 Department of Medical Oncology, Istanbul Onkoloji Hospital, Istanbul 34846, Turkey; 6 Department of Medical Oncology, Medipol Bahcelievler Hospital, Istanbul 34196, Turkey

**Keywords:** high-grade serous ovarian carcinoma, chemotherapy response score, neoadjuvant chemotherapy, interval debulking surgery, pathologic response, recurrence-free survival, overall survival

## Abstract

*Background and Objectives*: In advanced high-grade serous tubo-ovarian carcinoma (HGSOC), the chemotherapy response score (CRS) assesses histopathologic response to neoadjuvant chemotherapy (NACT). CRS1 and CRS2 are commonly grouped for similar prognostic outcomes, but whether partial response carries prognostic value and which factors predict pathologic response remain unclear. This multicenter study evaluated predictors of pathologic response and the association of CRS with recurrence-free survival (RFS) and overall survival (OS) after NACT followed by interval debulking surgery (IDS). *Materials and Methods*: We retrospectively analyzed 157 patients with FIGO stage IIIC-IV HGSOC treated with NACT followed by IDS between 2015 and 2021. Pathologic response was defined as CRS2–3 versus CRS1; logistic regression identified predictors of response. Survival was assessed using Kaplan–Meier analysis, and Cox regression evaluated factors associated with RFS and OS. Secondary analyses evaluated the conventional CRS1–2 versus CRS3 classification, direct CRS3-versus-CRS2 contrasts, and ordinal trends for RFS and OS. *Results*: CRS1, CRS2, and CRS3 were observed in 33 (21.0%), 97 (61.8%), and 27 (17.2%) patients, respectively. Median RFS was 10.4, 16.4, and 24.1 months for CRS1, CRS2, and CRS3, respectively (*p* < 0.001). Median OS was 21.2, 39.1, and 47.0 months, respectively (*p* < 0.001). In multivariate Cox analysis, CRS2 and CRS3 were independently associated with better RFS (HR 0.36 and 0.24) and OS (HR 0.48 and 0.41) compared with CRS1. In the conventional CRS1–2 versus CRS3 model, CRS3 remained associated with lower recurrence risk after multivariate adjustment (HR 0.60; *p* = 0.042), but not OS (HR 0.74; *p* = 0.354). Direct CRS3-versus-CRS2 contrasts were nonsignificant for RFS and OS (*p* = 0.131 and *p* = 0.628), whereas ordinal trends toward lower hazards were significant for both endpoints (*p* = 0.001 and *p* = 0.041). A higher CA-125 decrease rate (OR 1.04) and more than three NACT cycles (OR 4.89) independently predicted pathologic response, whereas FIGO stage IV predicted a lower response (OR 0.15). *Conclusions*: Partial pathological response (CRS2) was independently associated with improved RFS and OS compared with no or minimal response (CRS1), suggesting that prognostic information is not confined to complete or near-complete pathological response. The conventional CRS1–2 versus CRS3 classification remained independently prognostic for RFS after multivariate adjustment but did not reach statistical significance for OS, while direct CRS3-versus-CRS2 contrasts were nonsignificant for both outcomes. Thus, separate evaluation of CRS2 may provide additional RFS stratification within CRS1–2. However, the incremental contribution of CRS3 beyond CRS2 could not be established in the present cohort and requires validation in larger cohorts.

## 1. Introduction

Globally, ovarian cancer is the eighth most common cancer in women. High-grade serous ovarian carcinoma (HGSOC) is one of the most aggressive and most common types. Approximately 70% of cases are diagnosed at an advanced stage, and HGSOC accounts for the majority of these [1]. The standard management for newly diagnosed cases is primary debulking surgery aimed at removing all visible tumors, followed by an adjuvant chemotherapy regimen containing carboplatin and paclitaxel [2].

It is not always possible to achieve complete cytoreduction with primary debulking surgery in advanced stages, and suboptimal cytoreduction may be unavoidable. When complete cytoreduction with primary surgery is not feasible, neoadjuvant chemotherapy (NACT) followed by interval debulking surgery (IDS) is the preferred treatment choice. NACT has gained wider adoption because it enables improved cytoreduction outcomes with lower surgical morbidity than upfront surgery in patients with unresectable disease [3,4]. In terms of recurrence-free survival (RFS) and overall survival (OS), recent trials have demonstrated that NACT followed by IDS may be non-inferior to primary debulking surgery followed by adjuvant chemotherapy [5]. Complete cytoreduction is the most important prognostic factor in advanced-stage ovarian cancer, and it has been shown that neoadjuvant chemotherapy followed by IDS may increase the likelihood of achieving complete cytoreduction in selected patients [6].

The chemotherapy response score (CRS) system has been developed to assess the pathologic response to neoadjuvant chemotherapy. According to this scoring system, chemotherapy response is divided into three groups: CRS1 indicates no/minimal response, CRS2 indicates partial response, and CRS3 indicates complete/near-complete pathological response [7]. In addition, a multicenter retrospective study found that overall survival was higher among patients with complete or near-complete responses, regardless of the number of chemotherapy cycles [8]. CRS3 was associated with significantly improved progression-free survival compared with CRS1 and CRS2 [9].

Some previous studies found similar prognostic outcomes for the CRS1 and CRS2 groups, and these findings provided the basis for the commonly used grouping of CRS1 and CRS2 into a single non-complete-response category for comparison with CRS3 [7,9,10]. However, other studies have reported significant survival differences between CRS1 and CRS2. Considering that CRS2 represents a histopathological response, even if not a complete response, combining CRS1 and CRS2 may mask the prognostic contribution of partial response. Therefore, in our study, CRS2–3 was compared with CRS1, which represents no or minimal response, to evaluate the prognostic value of any appreciable histopathological response [11,12]. Additionally, the associations between the number of NACT cycles and survival outcomes, and between the CA-125 decrease rate and pathological response, have been reported inconsistently across cohorts. Moreover, the increasing use of maintenance therapies, particularly poly ADP-ribose polymerase (PARP) inhibitors and anti-angiogenic agents, has improved long-term outcomes in selected molecular subgroups and may confound the interpretation of OS when evaluating the prognostic impact of neoadjuvant chemotherapy response [13,14,15].

To address these gaps, this multicenter study aimed to evaluate predictors of pathologic response according to CRS and to assess the association of CRS with survival outcomes after NACT and IDS in International Federation of Gynecology and Obstetrics (FIGO) stage IIIC-IV HGSOC. Because the primary survival analyses evaluated CRS1, CRS2, and CRS3 as separate categories, secondary analyses were planned to assess whether the conventional CRS1–2 versus CRS3 grouping retained prognostic discrimination and to directly compare CRS2 with CRS3.

## 2. Materials and Methods

### 2.1. Study Design and Patient Selection

**Figure 1:**
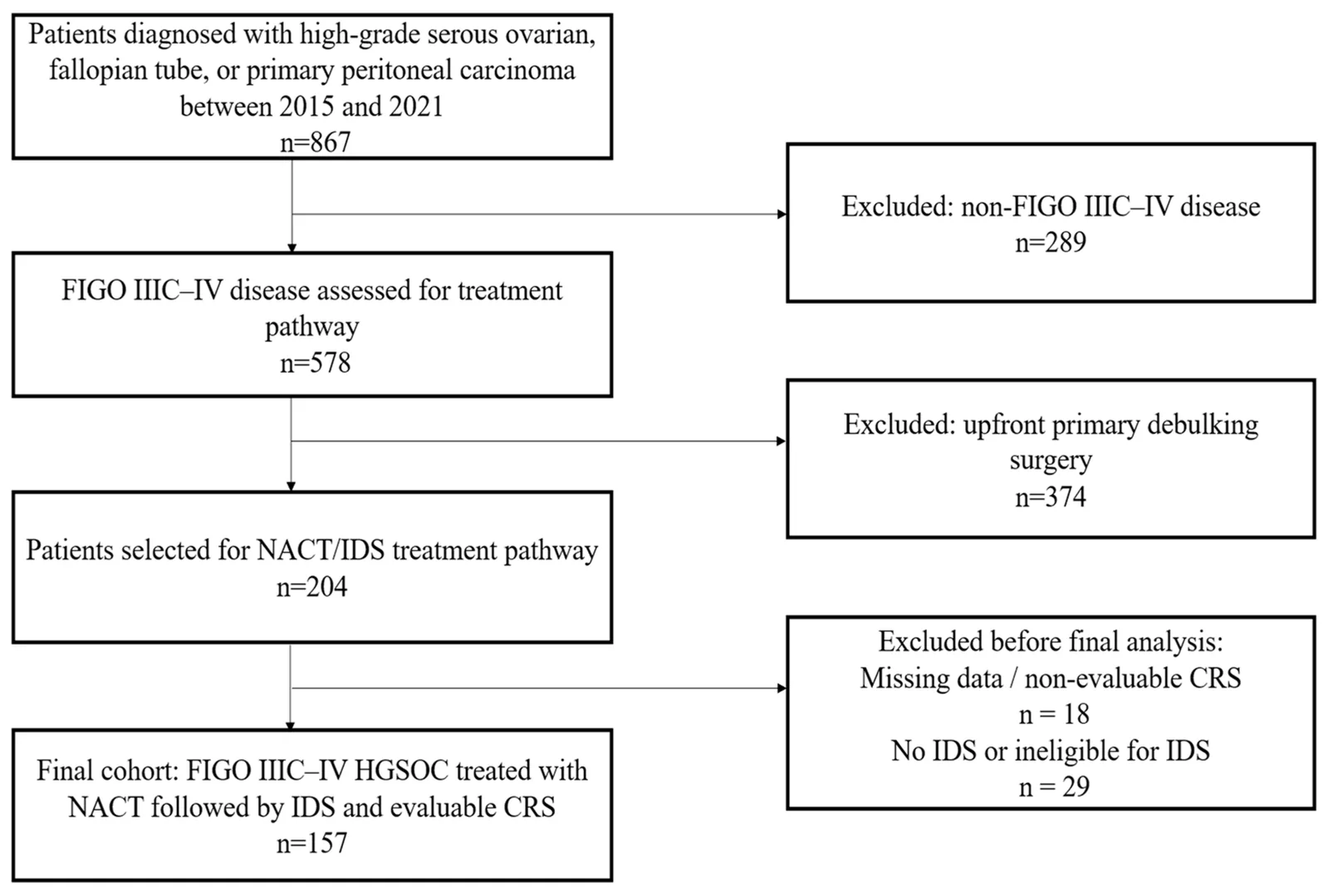
Flowchart of patient selection and study inclusion criteria.

We retrospectively analyzed the medical records of patients diagnosed with HGSOC between 2015 and 2021 in two oncology centers. A total of 157 patients were enrolled according to the following inclusion criteria: histologically confirmed diagnosis of high-grade serous carcinoma of the ovary, primary peritoneal, or fallopian tube, FIGO stage IIIC-IV disease, treatment with neoadjuvant chemotherapy followed by IDS, age ≥ 18 years, Eastern Cooperative Oncology Group (ECOG) performance status 0–2. Exclusion criteria were: non-HGSOC histology, patients who underwent upfront primary debulking surgery, patients who did not proceed to surgery regardless of reason (disease progression on chemotherapy, persistent unresectability, medical comorbidity, or patient refusal), and insufficient pathological material for CRS evaluation (Figure 1). Neoadjuvant chemotherapy was recommended for the patients in whom upfront complete cytoreduction was deemed not feasible by a multidisciplinary tumor board, based on imaging findings and clinical assessment.

The study was conducted in accordance with the Declaration of Helsinki and the guidelines for Good Clinical Practice. The study protocol was approved by the Clinical Research Ethics Committee of Istanbul Medeniyet University Göztepe Training and Research Hospital (Approval No: 2022/0713, Date: 7 December 2022). Due to the retrospective nature of the study, anonymized data were used in accordance with institutional ethical approval.

### 2.2. Data Collection and Procedures

Baseline clinical and demographic characteristics of the eligible patients were collected. The date of diagnosis, serum CA-125 levels (U/mL) at diagnosis and before IDS, germline BRCA mutation status, neoadjuvant chemotherapy regimen details (duration, dose, frequency, and number), FIGO stage, cytoreduction status, and CRS were also collected. The neoadjuvant chemotherapy regimen was paclitaxel 80mg/m^2^ and carboplatin area under the curve (AUC) 2 on days 1, 8, and 15 of a 21-day cycle, or paclitaxel 175mg/m^2^ and carboplatin AUC 5 on day 1 of a 21-day cycle. The CA-125 decrease rate was calculated by [(baseline CA-125 − pre-IDS CA-125)/baseline CA-125] × 100. Initial CA-125 levels were categorized using a cut-off of 1000 U/mL.

Resectability and radiologic response to neoadjuvant chemotherapy were evaluated by the tumor board, which included the gynecological oncology senior consultant, medical oncologist, radiologist, and pathologist, after initial cycles of chemotherapy, generally after 3 or 4 cycles, based on Response Evaluation Criteria in Solid Tumors version 1.1 (RECIST 1.1) radiologic assessment and clinical operability criteria [16]. As a result of the patient-based evaluation, neoadjuvant chemotherapy was extended to 5 or 6 cycles in patients who did not achieve resectability after initial cycles, according to the tumor board. IDS was performed in patients who achieved disease control per RECIST 1.1 and were deemed operable by the tumor board. Experienced oncological surgeons performed all surgical procedures.

Surgery aimed to obtain complete cytoreduction. Complete gross cytoreduction was defined as the absence of visible disease (R0). Optimal cytoreduction was defined as a residual disease with a maximum tumor diameter of ≤1 cm, and suboptimal cytoreduction was determined as a residual disease with a maximum tumor diameter of >1 cm [17,18,19]. An expert gynecologic oncology pathologist in each participating institution performed the pathology examination for CRS. CRS was assessed on omental tumor tissue obtained at IDS. CRS1 was defined as no response, CRS2 was defined as a partial response, and CRS3 was defined as a complete or nearly complete response.

In most patients after IDS, adjuvant chemotherapy was administered to complete a total of six chemotherapy cycles. Accordingly, patients who received three or four cycles of neoadjuvant chemotherapy generally received three or two postoperative cycles, respectively. In selected patients who had already received six neoadjuvant cycles before IDS, two or three additional postoperative chemotherapy cycles were administered at the discretion of the treating physician and multidisciplinary tumor board. All patients were followed up for disease recurrence in accordance with recent guidelines [2,20]. During adjuvant chemotherapy, physical examination and serum CA-125 levels were assessed every cycle, and imaging was performed every 3 cycles. In patients with clinical progression findings, earlier imaging was performed. Follow-up visits were made after adjuvant chemotherapy. Physical examination and CA-125 monitoring were performed every 3 months, and radiological imaging was performed every 6 months for the first 2 years. After that, physical examination and CA-125 monitoring were performed every 6 months, and radiological imaging was performed annually for 2–5 years.

Recurrence-free survival (RFS) and OS were examined. RFS was defined as the time from the IDS date to confirmed radiologic recurrence or death from any cause, and OS was defined as the time from the first diagnosis date to the date of death from any cause or the last follow-up time. Radiologic recurrence was defined according to RECIST 1.1.

### 2.3. Statistical Analysis

Qualitative variables were presented as numbers and percentages; continuous variables with a normal distribution were presented as mean ± SD; and continuous variables without a normal distribution were presented as medians and ranges. Differences in categorical variables were evaluated using the Chi-square test or Fisher’s exact test. Normally distributed data were analyzed using Student’s t-test, and non-normally distributed data were analyzed using the Mann–Whitney U test. Continuous variables were compared across the three CRS groups using one-way analysis of variance (ANOVA) for normally distributed variables and the Kruskal–Wallis test for non-normally distributed variables. Kaplan–Meier survival curves and log-rank tests were used to compare RFS and OS across the three CRS categories. In a secondary analysis, RFS and OS were also compared using the conventional binary classification of CRS1–2 versus CRS3. Follow-up time was determined using the reverse Kaplan–Meier method.

Univariate and multivariate Cox proportional hazards regression models and logistic regression were constructed to identify independent predictors of pathologic response, RFS, and OS. As part of the secondary analysis, the conventional CRS1–2 versus CRS3 classification was additionally evaluated in separate univariate and multivariate Cox models for RFS and OS, with CRS1–2 as the reference group. To ensure comparability with the primary three-category analyses, the multivariate binary models included the same outcome-specific covariates: age, cytoreduction status, and FIGO stage for RFS and age, ECOG performance status, cytoreduction status, and FIGO stage for OS. Direct CRS2-versus-CRS3 contrasts were obtained by re-parameterizing the univariate and multivariate three-category Cox models with CRS2 as the reference category, without excluding patients with CRS1. A formal test for linear trend across CRS categories was also performed by entering CRS as an ordinal variable coded as 1, 2, and 3 in univariate and multivariate Cox models, using the same outcome-specific covariates. The proportional hazards assumption was assessed using time-by-covariate interaction terms. In logistic regression, CRS2–3 versus CRS1, corresponding to the presence/absence of pathological response, was used as the dependent variable. The candidate covariates for pathologic response analyses were: age at initial diagnosis, ECOG performance status, BRCA mutation status, FIGO stage, initial CA-125 levels, CA-125 decrease rate, chemotherapy frequency (weekly or 3-weekly), and number of neoadjuvant chemotherapy cycles. The candidate covariates for RFS, OS analyses were: age at initial diagnosis, ECOG performance status, pathologic response (CRS1, CRS2, CRS3), cytoreduction status, FIGO stage, chemotherapy frequency (weekly or 3-weekly), and number of neoadjuvant chemotherapy cycles. ECOG was analyzed as a binary variable, referred to as ECOG 0 and ECOG 1–2 in Cox regression analyses. Variables with statistical significance (*p* < 0.05) or clinical relevance were included in the multivariate model. Results are reported as ORs or HRs with 95% CIs. Statistical analyses were performed with SPSS software version 22.0 (SPSS Inc., Chicago, IL, USA), and a *p*-value <0.05 was considered statistically significant.

## 3. Results

A total of 157 patients who received neoadjuvant chemotherapy were enrolled in our study. They were divided into groups according to the CRS results. Thirty-three patients (21%) were included in the CRS1 group, 97 (61.8%) in the CRS2 group, and 27 (17.2%) in the CRS3 group. The clinical and treatment-related characteristics are presented in Table 1. The mean age of the patients was 61.59 ± 11.24 years. The FIGO stage IV rates were 63.6%, 17.5%, and 25.9% in the CRS1, CRS2, and CRS3 groups, respectively (*p* < 0.001). The complete gross cytoreduction (R0) rate across the entire group was 74.5%. In the CRS1, CRS2, and CRS3 groups, these rates were 24.2%, 86.6%, and 92.6%, respectively (*p* < 0.001). BRCA mutation status was assessed in 89 of 157 patients (56.7%), and 25 (15.9%) harbored a deleterious BRCA mutation. The median initial CA-125 level was 1480 U/mL (range, 29–18,125 U/mL), 59.2% of patients had levels > 1000 U/mL, and the median CA-125 decrease rates were CRS1: 78%, CRS2: 95%, and CRS3: 96%, respectively (*p* < 0.001). Of the 157 patients, 40 (25.5%) received a weekly carboplatin–paclitaxel regimen, and 117 (74.5%) received a three-weekly carboplatin–paclitaxel regimen. According to the number of neoadjuvant cycles, two cycles accounted for 3.8%, three cycles for 64.3%, four cycles for 23.6%, five cycles for 5.7%, and six cycles for 2.5%. There was no significant difference between CRS groups in the characteristics described above (*p* > 0.05), except for the FIGO stage, the CA-125 decrease rate, and the cytoreduction type.

**Figure 2:**
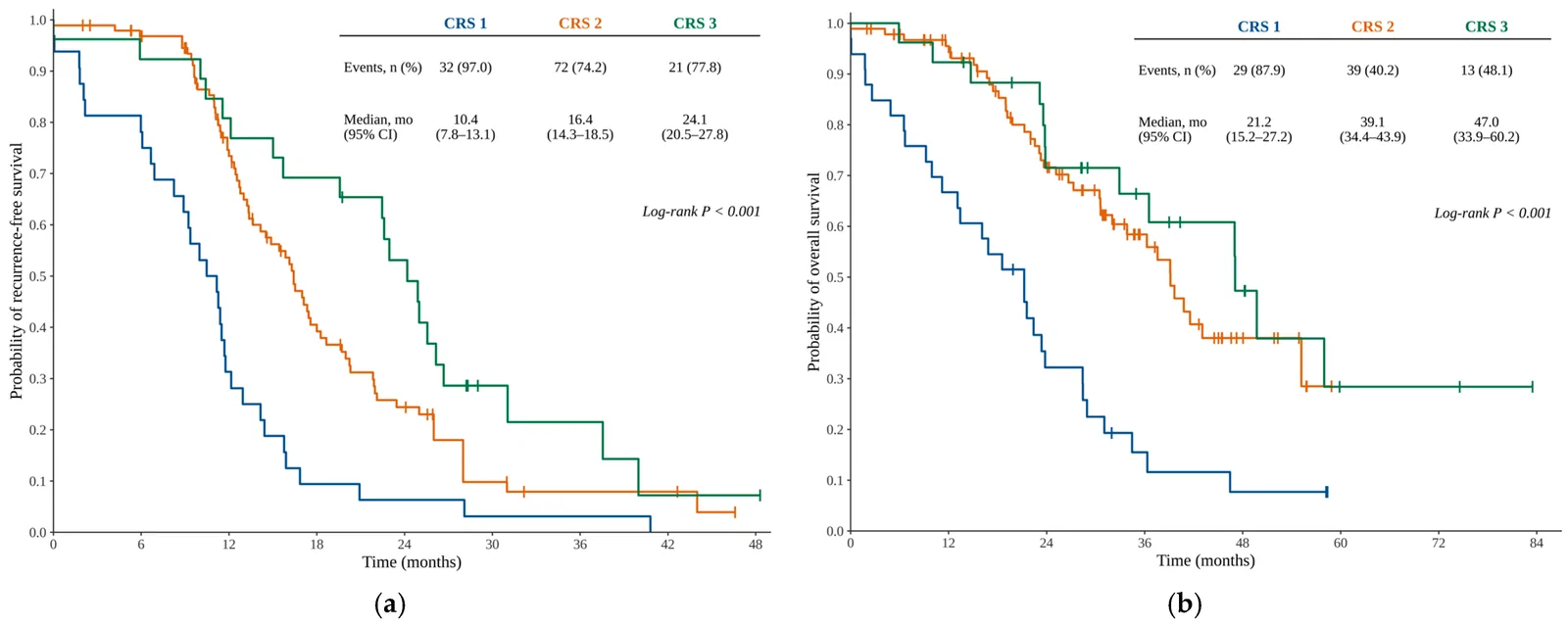
Kaplan–Meier survival curves according to chemotherapy response score. (**a**) Recurrence-free survival: median RFS was 10.4 months for CRS1, 16.4 months for CRS2, and 24.1 months for CRS3; log-rank *p* < 0.001. (**b**) Overall survival: median OS was 21.2 months for CRS1, 39.1 months for CRS2, and 47.0 months for CRS3; log-rank *p* < 0.001. CRS: Chemotherapy response score.

The median follow-up was 40.3 months (95% CI 30.9–49.9 months). During follow-up, 125 (79.6%) recurrence or death events occurred. The median RFS was 10.4 months (95% CI 7.8–13.1), 16.4 months (95% CI 14.3–18.5), and 24.1 months (95% CI 20.5–27.8) in the CRS1, CRS2, and CRS3 groups, respectively (*p* < 0.001) (Figure 2a). OS was also analyzed across CRS groups. A total of 81 deaths (51.6%) were recorded for OS analysis. The median OS was 21.2 months (95% CI 15.2–27.2), 39.1 months (95% CI 34.4–43.9), and 47 months (95% CI 33.9–60.2) in the CRS1, CRS2, and CRS3 groups, respectively (*p* < 0.001) (Figure 2b). In the secondary analysis, using the conventional binary classification CRS1–2 versus CRS3, the median RFS was 14.1 months (95% CI 11.8–16.5) in the CRS 1–2 group and 24.1 months (95% CI 20.5–27.8) in the CRS 3 group (*p* = 0.007). The median OS was 31 months (95% CI 24.3–37.8) in the CRS 1–2 group and 47 months (95% CI 33.9–60.2) in the CRS 3 group (*p* = 0.085). In univariate Cox regression analysis, CRS3 was associated with a lower risk of recurrence than CRS1–2 (HR 0.53, 95% CI 0.33–0.85; *p* = 0.008), whereas its association with OS was not statistically significant (HR 0.59, 95% CI 0.33–1.08; *p* = 0.089).

Univariate and multivariate logistic regression analyses were performed to identify factors predicting pathologic response, defined as CRS2 and CRS3 versus CRS1, representing the presence versus absence of pathologic response. The results are summarized in Table 2. A higher CA-125 decrease rate per 1% OR: 1.04 (95% CI 1.02–1.07; *p* < 0.001) and receiving more than three NACT cycles OR: 4.89 (95% CI 1.36–17.54; *p* = 0.015) were independently associated with improved pathologic response, and FIGO stage IV OR: 0.15 (95% CI 0.06–0.39; *p* < 0.001) was independently associated with reduced pathologic response.

Factors predicting RFS were evaluated with Cox regression. The proportional hazards assumption was met for the variables in both the RFS and OS Cox models, as assessed using time-by-covariate interaction terms (*p* = 0.156 and *p* = 0.329, respectively). In multivariate analysis, compared with the CRS1 group, CRS2 HR 0.36 (95% CI 0.19–0.67; *p* = 0.001) and CRS3 HR 0.24 (95% CI 0.12–0.51; *p* < 0.001) were significantly associated with lower recurrence risk. In univariate analyses, suboptimal cytoreduction HR 3.01 (95% CI 1.48–6.10; *p* = 0.002) was significantly associated with worse RFS; it was not significant in the multivariate analysis. Age, cytoreduction status, and FIGO stage were not significant in the multivariate analysis. Factors predicting OS were also evaluated with Cox regression. In the univariate Cox analyses, age, ECOG, CRS groups, cytoreduction status, and FIGO stage were statistically significant. In the multivariate OS analysis, age was independently associated with worse OS (HR 1.05, 95% CI 1.01–1.08; *p* = 0.002). Compared with the CRS1 group, CRS2 HR 0.48 (95% CI 0.25–0.93; *p* = 0.029) and CRS3 HR 0.41 (95% CI 0.18–0.93; *p* = 0.033) were independently associated with improved OS. Suboptimal cytoreduction was associated with worse OS HR 3.04 (95% CI 1.07–8.69; *p* = 0.036). ECOG performance status and the FIGO stage were significant in the univariate analysis but not in the multivariate analysis. The chemotherapy regimen and the number of neoadjuvant chemotherapy cycles were not significantly associated with either RFS or OS in univariate Cox analysis. The multivariate analysis results for RFS and OS are detailed in Table 3.

To permit direct comparison with the primary three-category model, the conventional CRS1–2 versus CRS3 classification was additionally evaluated using multivariate Cox models with the same outcome-specific covariates (Table A1). CRS3 remained independently associated with a lower recurrence risk compared with CRS1–2 (adjusted HR 0.60, 95% CI 0.37–0.98; *p* = 0.042). In contrast, its adjusted association with OS was not statistically significant (adjusted HR 0.74, 95% CI 0.39–1.40; *p* = 0.354). In the re-parameterized three-category models using CRS2 as the reference category (Table A2), the direct CRS3-versus-CRS2 contrast was not statistically significant for RFS in either the univariate analysis (HR 0.64, 95% CI 0.39–1.04; *p* = 0.073) or the multivariate analysis (adjusted HR 0.68, 95% CI 0.41–1.12; *p* = 0.131). Similarly, no statistically significant CRS3-versus-CRS2 contrast was observed for OS in the univariate analysis (HR 0.83, 95% CI 0.44–1.57; *p* = 0.568) or the multivariate analysis (adjusted HR 0.85, 95% CI 0.44–1.63; *p* = 0.628). When CRS was entered as an ordinal variable, each one-category increase was associated with lower hazards of recurrence in both the univariate (HR 0.49, 95% CI 0.36–0.66; *p* < 0.001) and multivariate models (adjusted HR 0.53, 95% CI 0.36–0.77; *p* = 0.001). Significant linear trends were also observed for OS in the univariate (HR 0.47, 95% CI 0.33–0.67; *p* < 0.001) and multivariate models (adjusted HR 0.64, 95% CI 0.42–0.98; *p* = 0.041) (Table A3).

## 4. Discussion

In our study, patients diagnosed with FIGO Stage IIIC-IV HGSOC were divided into three groups based on their CRS levels after interval debulking following neoadjuvant chemotherapy. The clinical and treatment-related characteristics of the groups were similar except for FIGO stage, CA-125 decrease rate, and cytoreduction type. The presence of a pathological response in the CRS2 and CRS3 groups was compared with its absence in the CRS1 group. FIGO stage IV was associated with a worse pathological response, while a higher CA-125 decrease rate and receiving more than three cycles of neoadjuvant chemotherapy were found to be associated with a better pathological response. Kaplan–Meier analyses showed that higher CRS was associated with better RFS and OS. CRS was also independently associated with both RFS and OS in multivariate Cox regression. Age and suboptimal cytoreduction were independently associated with worse OS. ECOG performance status and the FIGO stage were not independently associated with OS in multivariate Cox models. Chemotherapy frequency and the number of neoadjuvant chemotherapy cycles were not associated with RFS and OS in univariate analyses.

The median RFS was 10.4 months in CRS1, 16.4 months in CRS2, and 24.1 months in CRS3. In multivariate Cox regression analysis, both CRS2 and CRS3 were associated with significantly improved RFS compared with CRS1 (CRS2: HR 0.36, 95% CI 0.19–0.67; *p* = 0.001, CRS3: HR 0.24, 95% CI 0.12–0.51; *p* < 0.001), indicating that recurrence risk was lower in both groups relative to CRS1. However, these comparisons alone do not demonstrate statistically significant progressive separation across all three CRS categories. Cohen et al., in their meta-analysis, compared CRS1–2 vs. CRS3. They evaluated CRS1 and CRS2 together. CRS3 was associated with a significantly improved progression-free survival (PFS) compared to CRS1–2 [9]. Similarly, Ditzel et al. combined CRS1 and CRS2; they found no difference in PFS between the groups and did not demonstrate that CRS2 had independent prognostic value [10]. However, in our study, a significantly better RFS was observed in CRS2 than in CRS1. This finding suggests that a pathological response may be valuable at every level, even if a complete response is not achieved. This finding also supported our choice to define pathological response as CRS2–3 versus CRS1 in the logistic regression model. In Michaan et al.’s three-group analysis, only CRS3 was significant for PFS, whereas CRS2 did not reach statistical significance [21].

In both univariate and multivariate OS analyses, CRS was significantly associated with OS. In the multivariate analysis, CRS2 (HR 0.48, 95% CI 0.25–0.93; *p* = 0.029) and CRS3 (HR 0.41, 95% CI 0.18–0.93; *p* = 0.033) were associated with significantly improved OS compared with CRS1. For OS, the HR values of CRS2 and CRS3 were close, and their confidence intervals overlapped, indicating limited prognostic separation between these groups in our cohort. Consistently, the conventional CRS1–2 versus CRS3 comparison was not statistically significant for OS. While CRS was independently associated with OS in our study, its prognostic role for OS has been more inconsistent in the literature than that of RFS. Studies by Michaan et al., Ditzel et al., and Lawson et al. found no significant association with OS. Even in Lee et al.’s multicenter study, the OS association did not reach significance [10,13,21,22]. However, the meta-analysis by Cohen et al. found a significant association between CRS3 and OS [9]. The significant result for CRS in the OS analysis may be partly attributable to evaluating CRS across three separate groups, which allowed the prognostic contribution of CRS2 to be assessed separately from that of CRS1. Additionally, by including the FIGO stage in the multivariate analysis, we controlled for stage-dependent variation in OS. Although FIGO stage IV distribution was imbalanced across CRS groups, CRS retained independent prognostic significance after adjustment for FIGO stage. This suggests that the relationship between CRS and survival is unlikely to be explained solely by the stage distribution. Age was also independently associated with worse OS in the multivariate model (HR 1.05 per year, 95% CI 1.01–1.08; *p* = 0.002). ECOG performance status, which was significant in the univariate OS analysis (HR 1.86, *p* = 0.007), completely lost its significance in the multivariate analysis (HR 1.01, *p* = 0.980). This suggests that the univariate association of ECOG with OS may be partly explained by age and other covariates included in the multivariate model. OS reflects a long process encompassing chemotherapy and maintenance therapies after NACT. Since we did not have maintenance treatment data in our study, this confounding factor could not be controlled for. Although CRS was independently associated with OS in the multivariate analysis, these results should be interpreted with caution, given potential residual confounding from factors not captured in our dataset, particularly maintenance therapy and post-recurrence treatment strategies.

The binary comparison of CRS2–3 and CRS1 is clinically important. Unlike the conventional CRS1–2 versus CRS3 comparison, which primarily identifies patients with complete or near-complete responses, this comparison evaluates whether any appreciable histopathological response carries prognostic value compared with no or minimal response.

To determine whether the conventional classification retains prognostic discrimination in our cohort, RFS and OS were additionally compared using the CRS1–2 versus CRS3 grouping. The conventional classification retained prognostic discrimination for RFS in both the univariate analysis (HR 0.53, 95% CI 0.33–0.85; *p* = 0.008) and the multivariate analysis using the same covariates as the primary three-category model (adjusted HR 0.60, 95% CI 0.37–0.98; *p* = 0.042), demonstrating that the association remained statistically significant after adjustment for age, cytoreduction status, and FIGO stage. However, its association with OS did not reach statistical significance in either the univariate analysis (HR 0.59, 95% CI 0.33–1.08; *p* = 0.089) or the multivariate analysis (adjusted HR 0.74, 95% CI 0.39–1.40; *p* = 0.354). These findings indicate that, for RFS, our three-category analysis may provide an additional layer of prognostic stratification within the conventionally defined CRS1–2 group. For OS, however, the discrimination gained between CRS1 and CRS2 in our cohort appears to have come, at least in part, at the expense of the discrimination between CRS2 and CRS3 captured by the conventional grouping. This OS comparison in our cohort is consistent with Böhm et al. and Ditzel et al., both of whom reported nonsignificant trends toward longer OS with CRS3, but differs from the meta-analysis by Cohen et al., which demonstrated significantly improved OS for CRS3 compared with CRS1–2 [7,9,10].

Because both CRS2 and CRS3 were independently associated with improved RFS and OS compared with CRS1, we further examined whether this represented statistically significant progressive separation across all three categories or was primarily driven by their comparisons with CRS1. Two complementary approaches were used. First, the three-category models were re-parameterized with CRS2 as the reference category to obtain a direct CRS3-versus-CRS2 contrast; this comparison did not reach statistical significance for RFS (univariate HR 0.64, 95% CI 0.39–1.04; *p* = 0.073; multivariate HR 0.68, 95% CI 0.41–1.12; *p* = 0.131) or OS (univariate HR 0.83, 95% CI 0.44–1.57; *p* = 0.568; multivariate HR 0.85, 95% CI 0.44–1.63; *p* = 0.628). Second, CRS was modeled as an ordinal covariate to formally test for a linear trend across categories; significant trends toward lower hazards with increasing CRS were observed for both RFS (multivariate HR 0.53 per one-category increase, 95% CI 0.36–0.77; *p* = 0.001) and OS (multivariate HR 0.64, 95% CI 0.42–0.98; *p* = 0.041). Taken together, these analyses support an overall ordered association between CRS and survival but do not demonstrate statistically significant prognostic separation between CRS2 and CRS3. The relatively small CRS3 subgroup and wide confidence intervals may have limited the precision of the direct comparisons. Nevertheless, CRS2 remained independently associated with improved RFS and OS compared with CRS1, suggesting that a partial pathological response may carry prognostic information relative to no or minimal response. This finding should not be interpreted as evidence of prognostic equivalence between CRS2 and CRS3; the incremental prognostic contribution of CRS3 beyond CRS2 could not be established in the present cohort.

CRS1 may represent a patient group with chemotherapy-resistant tumor biology. However, the higher proportion of FIGO stage IV disease in the CRS1 group may have contributed to poorer survival and accentuated the prognostic separation between CRS1 and CRS2. In contrast, all CRS groups were managed within the same general treatment framework of platinum-taxane-based NACT followed by IDS, and CRS was assessed on omental specimens using the same three-tier Böhm system. Therefore, no systematic difference in treatment context or CRS assessment was identified that would selectively account for the survival advantage observed in the CRS2 group, although residual confounding related to cohort composition cannot be excluded. Against this background, the survival advantage observed in the CRS2 group suggests that a partial pathological response may carry additional prognostic information, particularly for RFS, for which the conventional CRS1–2 versus CRS3 classification also retained prognostic discrimination. For OS, this finding should be interpreted more cautiously because the conventional grouping did not reach statistical significance in our cohort. This interpretation is supported by Lee et al., who reported a significant PFS difference between CRS1 and CRS2 and observed that CRS2 outcomes were closer to those of CRS3, and by Santhamma et al., who demonstrated significantly better PFS and OS for CRS2 than for CRS1 [11,12]. This finding suggests that grouping CRS1 and CRS2 together may mask the clinical signal of CRS2; it should be considered not as definitive evidence warranting changes in clinical management, but as a hypothesis-forming finding that needs to be confirmed in prospective studies.

FIGO stage IV was the strongest independent negative predictor of pathological response (OR: 0.15, 95% CI 0.06–0.39). FIGO stage IV encompasses extraperitoneal spread and may be associated with a lower response to chemotherapy than peritoneal disease. Petrillo et al. also identified FIGO stage IV as the only independent negative predictor of complete pathological response [23]. As the CA-125 decrease rate increases, the probability of a pathological response increases independently (OR: 1.04). This finding suggests that serological response reflects biological sensitivity to chemotherapy and can predict pathological response. In the study by Liang et al. investigating CRS3 markers, the post-NACT CA-125 reduction rate was identified as the strongest independent predictor [24]. Similar differences in the CA-125 decrease rate across CRS groups have been reported by Michaan et al. [21].

The pathological response rate was significantly higher in patients who received more than 3 cycles of neoadjuvant chemotherapy (OR: 4.89, 95% CI 1.36–17.54). Patients receiving more than 3 cycles may be those who did not progress after neoadjuvant chemotherapy and may have responded; that is, this may indicate that patients who could already respond received more cycles. This finding is inherent to our retrospective cohort. In addition, a 2023 meta-analysis showed that receiving more than 3 cycles was associated with modestly worse OS compared to ≤3 cycles, although no significant PFS difference was observed [25]. The vast majority of the >3 cycle group in our study (74%) received only 4 cycles. This suggests that treatment was extended in patients who were still responding, rather than in those who had failed to benefit from chemotherapy. Betrian et al. found that CRS was associated with survival, regardless of the number of NACT cycles, further reinforcing the concept that histological chemosensitivity, rather than treatment duration, drives prognosis [8]. Weekly or 3-weekly chemotherapy was not associated with RFS or OS. Therefore, it was not included in the main multivariate model.

Suboptimal cytoreduction was associated with worse RFS and OS in univariate analysis. In multivariate analysis, this association did not retain independent significance for RFS, whereas for OS, suboptimal cytoreduction remained independently associated with worse survival compared with complete cytoreduction. This finding supports the idea that macroscopic residual disease in patients undergoing IDS after NACT has a significant impact, particularly on long-term survival. In contrast, optimal cytoreduction did not differ significantly from complete cytoreduction. This finding suggests a close relationship between pathological response and the quality of cytoreduction. Suboptimal cytoreduction was achieved in 27% of CRS1 cases but in 0% of CRS3 cases. Therefore, part of the effect attributed to cytoreduction may partly reflect tumor biology and chemotherapy sensitivity. Phillips et al. similarly found no difference in OS between R0 and R1 resections, whereas R2 resections were associated with significantly poorer survival, consistent with the survival impact of gross residual disease observed in our univariate analysis [26]. In the EORTC 55971 and CHORUS studies, complete cytoreduction was identified as the most important predictor of OS in IDS [5,27].

Recent validation data suggest that the prognostic value of CRS may be attenuated in patients with BRCA1/2 mutations or those receiving PARP inhibitor maintenance. In a multicenter cohort of 466 patients, the PFS advantage associated with CRS3 in the overall population was not retained among patients with BRCA1/2 mutations or those receiving PARP inhibitor maintenance [13]. Therefore, incomplete BRCA1/2 data and the absence of information on homologous recombination deficiency (HRD) and maintenance therapy in our cohort may have affected the observed association between CRS and survival.

This study has several limitations. As a retrospective study, patient selection, treatment decisions, and follow-up processes were not protocol-driven, introducing a risk of selection bias and unmeasured confounding. Patients who did not proceed to IDS were excluded; therefore, the findings reflect a surgically selected subpopulation rather than the full NACT-treated population. The relatively small CRS3 group and the imbalance in FIGO stage IV rates across CRS groups represent potential sources of instability in multivariate HR estimates and residual confounding, respectively. BRCA mutation status was not tested in 43.3% of patients, precluding BRCA-stratified CRS analysis; however, BRCA status was not used to guide NACT administration in this cohort. Data on maintenance therapy and post-recurrence treatment strategies were not systematically recorded, limiting the interpretation of OS because these clinically relevant factors could not be controlled for. Although CRS was assessed by experienced gynecologic oncology pathologists, central pathology review was not performed. Interobserver variability therefore remains a potential source of misclassification, particularly when distinguishing CRS1 from CRS2. However, all assessments were based on omental specimens, for which CRS has demonstrated better reproducibility and more consistent prognostic performance than adnexal scoring [10]. Real-world radiologic assessment may have introduced interobserver variability. Moreover, standardized quantitative measures of baseline tumor burden and surgical complexity were not available. Variability in chemotherapy scheduling (weekly or every 3 weeks), the number of NACT cycles (2–6), and the number of adjuvant chemotherapy cycles reflect real-world practice but may also contribute to treatment heterogeneity. Nevertheless, the multicenter design improves external validity compared with a single-center study.

## 5. Conclusions

In the three-category model, CRS2 and CRS3 were independently associated with improved RFS and OS compared with CRS1, and formal trend analyses showed significant overall linear trends toward lower hazards with increasing CRS for both endpoints. However, the direct CRS3-versus-CRS2 contrasts did not reach statistical significance for either outcome. The conventional CRS1–2 versus CRS3 classification remained independently prognostic for RFS after multivariate adjustment but did not reach statistical significance for OS. Therefore, separate evaluation of CRS2 may provide additional RFS stratification within the conventionally defined CRS1–2 group, whereas its prognostic relevance for OS should be interpreted with caution. The incremental prognostic contribution of CRS3 beyond CRS2 could not be established in the present cohort. FIGO stage IV was associated with a lower pathological response, whereas the higher CA-125 decrease rate was associated with a higher pathological response. Suboptimal cytoreduction was associated with worse OS. Although receiving more than three NACT cycles was independently associated with pathological response, the number of NACT cycles was not associated with RFS or OS; therefore, this finding should be interpreted cautiously and should not be considered evidence of a direct survival benefit from extended NACT. OS may be influenced by uncontrolled confounding factors, including maintenance therapy and post-recurrence treatment strategies. These findings should be confirmed in prospective, large-scale studies incorporating BRCA1/2 mutation status, HRD status, maintenance therapy, post-recurrence treatment strategies, standardization of tumor burden assessment, and the surgical complexity score.

## Figures and Tables

**Table 1:** Clinical and treatment-related characteristics across CRS groups in study patients.

	CRS1 (n = 33) 21%	CRS2 (n = 97) 61.8%	CRS3 (n = 27) 17.2%	Overall (n = 157)	*p*-Value
Age, mean ± SD	63.85 ± 10.48	61.52 ± 11.22	59.07 ± 12.06	61.59 ± 11.24	0.263
ECOG (%)					0.233
0	20 (60.6)	51 (52.6)	18 (66.7)	89 (56.7)	
1	6 (18.2)	25 (25.8)	8 (29.6)	39 (24.8)	
2	7 (21.2)	21 (21.6)	1 (3.7)	29 (18.5)	
FIGO (%)					
IIIC	12 (36.4)	80 (82.5)	20 (74.1)	112 (71.3)	<0.001
IV	21 (63.6)	17 (17.5)	7 (25.9)	45 (28.7)	
BRCA mutation status (%)					
Mutant	4 (12.1)	17 (17.5)	4 (14.8)	25 (15.9)	0.160
Wild	14 (42.2)	44 (45.4)	6 (22.2)	64 (40.8)	
Unknown	15 (45.5)	36 (37.1)	17 (63.0)	68 (43.3)	
Initial CA-125 Levels (U/mL) median (range)	1230 (121–10,000)	1765 (29–18,125)	854 (57–14,538)	1480 (29–18,125)	0.347
<1000 (%)	14 (42.4)	36 (37.1)	14 (51.9)	64 (40.8)	0.378
>1000 (%)	19 (57.6)	61 (62.9)	13 (48.1)	93 (59.2)	
CA-125 decrease rate, per 1% (median %)	78%	95%	96%	94%	<0.001
Chemotherapy Regimen (%)					
Weekly	7 (21.2)	28 (28.9)	5 (18.5)	40 (25.5)	0.451
3-weekly	26 (78.8)	69 (71.1)	22 (81.5)	117 (74.5)	
Neoadjuvant Cycles (%)					
2	3 (9.1)	2 (2.1)	1 (3.7)	6 (3.8)	0.210
3	25 (75.8)	59 (60.8)	17 (63)	101 (64.3)	
4	3 (9.1)	26 (26.8)	8 (29.6)	37 (23.6)	
5	2 (6.1)	7 (7.2)	-	9 (5.7)	
6	-	3 (3.1)	1 (3.7)	4 (2.5)	
Cytoreduction Type					<0.001
Complete Cytoreduction	8 (24.2)	84 (86.6)	25 (92.6)	117 (74.5)	
Residual disease ≤ 1 cm	16 (48.5)	10 (10.3)	2 (7.4)	28 (17.8)	
Residual disease > 1 cm	9 (27.3)	3 (3.1)	-	12 (7.6)	

CRS: Chemotherapy response score, ECOG: Eastern Cooperative Oncology Group, FIGO: International Federation of Gynecology and Obstetrics.

**Table 2:** Logistic regression analysis of factors associated with pathologic response: CRS2–3 versus CRS1.

	Univariate		Multivariate	
	OR (95% CI)	*p*-Value	OR (95% CI)	*p*-Value
Age at initial diagnosis (y)	0.97 (0.94–1.01)	0.195		
ECOG				
0	1			
1	1.01 (0.41–2.94)	0.853		
2	1.75 (0.51–5.90)	0.367		
BRCA Mutation Status				
Mutant	1			
Wild	0.68 (0.2–2.31)	0.537		
Unknown	0.67 (0.2–2.26)	0.522		
FIGO				
IIIC	1		1	
IV	0.13 (0.05–0.31)	<0.001	0.15 (0.06–0.39)	<0.001
CA-125				
≤1000	1			
>1000	0.82 (0.50–2.37)	0.827		
CA-125 decrease rate (per 1%)	1.04 (1.02–1.06)	<0.001	1.04 (1.02–1.07)	<0.001
Chemotherapy Regimen				
3-weekly	1			
weekly	1.34 (0.53–3.40)	0.528		
Number of Cycles				
≤3	1		1	
>3	3.08 (1.11–8.54)	0.031	4.89 (1.36–17.54)	0.015

OR: Odds ratio, ECOG: Eastern Cooperative Oncology Group, FIGO: International Federation of Gynecology and Obstetrics, CI: confidence interval.

**Table 3:** Univariate and multivariate Cox regression analysis for RFS and OS.

	Recurrence-Free Survival				Overall Survival			
	Univariate		Multivariate		Univariate		Multivariate	
	*p*-Value	HR (95% CI)	HR (95% CI)	*p*-Value	HR (95% CI)	*p*-Value	HR (95% CI)	*p*-Value
Age, per year	1.01 (0.99–1.03)	0.078	1.01 (0.99–1.03)	0.084	1.04 (1.02–1.06)	<0.001	1.05 (1.01–1.08)	0.002
ECOG								
0	1				1		1	
1–2	1.22 (0.85–1.75)	0.277			1.86 (1.18–2.91)	0.007	1.01 (0.51–1.99)	0.980
Pathologic Response								
CRS1	1		1		1		1	
CRS2	0.40 (0.26–0.61)	<0.001	0.36 (0.19–0.67)	0.001	0.33 (0.20–0.54)	<0.001	0.48 (0.25–0.93)	0.029
CRS3	0.25 (0.15–0.45)	<0.001	0.24 (0.12–0.51)	<0.001	0.27 (0.14–0.53)	<0.001	0.41 (0.18–0.93)	0.033
Cytoreduction Type								
Complete	1		1		1		1	
Optimal	1.46 (0.95–2.26)	0.089	0.88 (0.50–1.56)	0.679	2.14 (1.29–3.54)	0.003	1.15 (0.60–2.20)	0.664
Suboptimal	3.01 (1.48–6.10)	0.002	1.32 (0.50–3.51)	0.569	6.09 (2.91–12.7)	<0.001	3.04 (1.07–8.69)	0.036
FIGO Stage								
IIIC	1		1		1		1	
IV	1.38 (0.93–2.05)	0.105	0.85 (0.50–1.41)	0.527	2.79 (1.75–4.44)	<0.001	1.45 (0.77–2.71)	0.243
Chemotherapy Regimen								
3-weekly	1				1			
weekly	1.08 (0.72–1.60)	0.716			1.52 (0.93–2.49)	0.092		
Number of Cycles								
≤3	1				1			
>3	1.02 (0.69–1.50)	0.925			1.04 (0.63–1.71)	0.866		

HR: Hazard ratio, RFS: recurrence-free survival, OS: overall survival, CRS: chemotherapy response score.

## Data Availability

Data is available from the corresponding author on reasonable request.

## Abbreviations

The following abbreviations are used in this manuscript:

HGSOC	High-grade serous tubo-ovarian carcinoma
NACT	Neoadjuvant chemotherapy
IDS	Interval debulking surgery
CRS	Chemotherapy response score
FIGO	International Federation of Gynecology and Obstetrics
RFS	Recurrence-free survival
OS	Overall survival
ECOG	Eastern Cooperative Oncology Group
HR	Hazard ratio
OR	Odds ratio
CI	Confidence interval
RECIST	Response Evaluation Criteria in Solid Tumors
SD	Standard deviation
BRCA	Breast cancer susceptibility gene
PARP	Poly ADP-ribose polymerase
HRD	Homologous recombination deficiency

## Appendix A

**Table A1:** Univariate and multivariate Cox regression analyses using the conventional CRS1–2 versus CRS3 classification.

	Recurrence-Free Survival				Overall Survival			
	Univariate		Multivariate		Univariate		Multivariate	
	HR (95% CI)	*p*-Value	HR (95% CI)	*p*-Value	HR (95% CI)	*p*-Value	HR (95% CI)	*p*-Value
Age, per year	1.01 (0.99–1.03)	0.078	1.01 (0.99–1.03)	0.087	1.04 (1.02–1.06)	<0.001	1.05 (1.02–1.09)	0.001
ECOG								
0	1				1		1	
1–2	1.22 (0.85–1.75)	0.277			1.86 (1.18–2.91)	0.007	0.83 (0.43–1.59)	0.569
Pathologic Response								
CRS1–2	1		1		1		1	
CRS3	0.53 (0.33–0.85)	0.008	0.60 (0.37–0.98)	0.042	0.59 (0.33–1.08)	0.089	0.74 (0.39–1.40)	0.354
Cytoreduction Type								
Complete	1		1		1		1	
Optimal	1.46 (0.95–2.26)	0.089	1.32 (0.83–2.10)	0.238	2.14 (1.29–3.54)	0.003	1.39 (0.77–2.50)	0.270
Suboptimal	3.01 (1.48–6.10)	0.002	2.75 (1.18–6.38)	0.019	6.09 (2.91–12.7)	<0.001	4.72 (1.84–12.14)	0.001
FIGO Stage								
IIIC	1		1		1		1	
IV	1.38 (0.93–2.05)	0.105	1.08 (0.68–1.72)	0.754	2.79 (1.75–4.44)	<0.001	1.72 (0.96–3.07)	0.067
Chemotherapy Regimen								
3-weekly	1				1			
weekly	1.08 (0.72–1.60)	0.716			1.52 (0.93–2.49)	0.092		
Number of Cycles								
≤3	1				1			
>3	1.02 (0.69–1.50)	0.925			1.04 (0.63–1.71)	0.866		

HR: Hazard ratio, CRS: chemotherapy response score.

**Table A2:** Univariate and multivariate Cox regression analyses using CRS2 as the reference category.

	Recurrence-Free Survival				Overall Survival			
	Univariate		Multivariate		Univariate		Multivariate	
	HR (95% CI)	*p*-Value	HR (95% CI)	*p*-Value	HR (95% CI)	*p*-Value	HR (95% CI)	*p*-Value
Age, per year	1.01 (0.99–1.03)	0.078	1.01 (0.99–1.03)	0.084	1.04 (1.02–1.06)	<0.001	1.05 (1.01–1.08)	0.002
ECOG								
0	1				1		1	
1–2	1.22 (0.85–1.75)	0.277			1.86 (1.18–2.91)	0.007	1.01 (0.51–1.99)	0.980
Pathologic Response								
CRS2	1		1		1		1	
CRS3	0.64 (0.39–1.04)	0.073	0.68 (0.41–1.12)	0.131	0.83 (0.44–1.57)	0.568	0.85 (0.44–1.63)	0.628
CRS1	2.50 (1.64–3.81)	<0.001	2.76 (1.49–5.11)	0.001	3.02 (1.86–4.90)	<0.001	2.05 (1.08–3.90)	0.029
Cytoreduction Type								
Complete	1		1		1		1	
Optimal	1.46 (0.95–2.26)	0.089	0.89 (0.50–1.56)	0.679	2.14 (1.29–3.54)	0.003	1.15 (0.61–2.19)	0.664
Suboptimal	3.01 (1.48–6.10)	0.002	1.33 (0.50–3.51)	0.569	6.09 (2.91–12.7)	<0.001	3.04 (1.08–8.60)	0.036
FIGO Stage								
IIIC	1		1		1		1	
IV	1.38 (0.93–2.05)	0.105	0.85 (0.51–1.42)	0.527	2.79 (1.75–4.44)	<0.001	1.45 (0.78–2.72)	0.243
Chemotherapy Regimen								
3-weekly	1				1			
weekly	1.08 (0.72–1.60)	0.716			1.52 (0.93–2.49)	0.092		
Number of Cycles								
≤3	1				1			
>3	1.02 (0.69–1.50)	0.925			1.04 (0.63–1.71)	0.866		

HR: Hazard ratio, :CRS: chemotherapy response score.

**Table A3:** Univariate and multivariate Cox regression analyses of the linear trend across CRS categories.

	Univariate HR (95% CI)	*p*-Value	Multivariate HR (95% CI)	*p*-Value
RFS	0.49 (0.36–0.66)	<0.001	0.53 (0.36–0.77)	0.001
OS	0.47 (0.33–0.67)	<0.001	0.64 (0.42–0.98)	0.041

CRS was entered as an ordinal variable coded 1, 2, and 3; therefore, the HR represents the change in hazard associated with each one-category increase in CRS. The multivariate RFS model was adjusted for age, cytoreduction status, and FIGO stage. The multivariate OS model was adjusted for age, ECOG performance status, cytoreduction status, and FIGO stage. HR: hazard ratio; CI: confidence interval; RFS: recurrence-free survival; OS: overall survival; CRS: chemotherapy response score.
